# Lack of survival advantage with autologous stem-cell transplantation in high-risk neuroblastoma consolidated by anti-G_D2_ immunotherapy and isotretinoin

**DOI:** 10.18632/oncotarget.6393

**Published:** 2015-11-26

**Authors:** Brian H. Kushner, Irina Ostrovnaya, Irene Y. Cheung, Deborah Kuk, Shakeel Modak, Kim Kramer, Stephen S. Roberts, Ellen M. Basu, Karima Yataghene, Nai-Kong V. Cheung

**Affiliations:** ^1^ Department of Pediatrics, Memorial Sloan Kettering Cancer Center, New York, NY 10065, USA; ^2^ Department of Epidemiology and Biostatistics, Memorial Sloan Kettering Cancer Center, New York, NY 10065, USA

**Keywords:** immunotherapy, anti-G_D2_ antibody, minimal residual disease, autologous stem-cell transplantation, pediatric oncology

## Abstract

Since 2003, high-risk neuroblastoma (HR-NB) patients at our center received anti-G_D2_ antibody 3F8/GM-CSF + isotretinoin – but not myeloablative therapy with autologous stem-cell transplantation (ASCT). Post-ASCT patients referred from elsewhere also received 3F8/GM-CSF + isotretinoin. We therefore accrued a study population of two groups treated during the same period and whose consolidative therapy, aside from ASCT, was identical. We analyzed patients enrolled in 1st complete/very good partial remission (CR/VGPR). Their event-free survival (EFS) and overall survival (OS) were calculated from study entry. Large study size allowed robust statistical analyses of key prognosticators including *MYCN* amplification, minimal residual disease (MRD), *FCGR2A* polymorphisms, and killer immunoglobulin-like receptor genotypes of natural killer cells. The 170 study patients included 60 enrolled following ASCT and 110 following conventional chemotherapy. The two cohorts had similar clinical and biological features. Five-year rates for ASCT and non-ASCT patients were, respectively: EFS 65% vs. 51% (*p* = .128), and OS 76% vs. 75% (*p* = .975). In multivariate analysis, ASCT was not prognostic and only MRD-negativity after two cycles of 3F8/GM-CSF correlated with significantly improved EFS and OS. Although a trend towards better EFS is seen with ASCT, OS is near identical. Cure rates may be similar, as close surveillance detects localized relapse and effective salvage treatments are applied. ASCT may not be needed to improve outcome when anti-G_D2_ immunotherapy is used for consolidation after dose-intensive conventional chemotherapy.

## INTRODUCTION

Since 2003, treatment of high-risk neuroblastoma (HR-NB) at Memorial Sloan Kettering Cancer Center (MSKCC) has included dose-intensive induction chemotherapy [1–3] ± 2nd -line therapy (i.e., additional cycles of chemotherapy) [4–7] if necessary to achieve complete/very good partial remission (CR/VGPR). Consolidation has comprised immunotherapy using anti-G_D2_ 3F8 monoclonal antibody (mAb) plus granulocyte-macrophage colony-stimulation factor (GM-CSF), isotretinoin, and local radiotherapy (RT) [8] – but not myeloablative therapy with autologous stem-cell transplantation (ASCT) which is standard elsewhere [9].

Our group adopted ASCT consolidation of HR-NB in the 1980s based on the concept that high-dose alkylators can overcome chemoresistance [10, 11]. Subsequently, after promising results with high-dose cyclophosphamide in induction [12], as well as with 3F8 in phase I and II trials [13, 14], we undertook a non-ASCT study using dose-intensive induction followed by consolidation with 3F8 [15] and local RT [16]. The 1999 report showing the advantage of ASCT in the landmark randomized Children's Cancer Group (CCG) study [17] prompted us to resume ASCT. In 2003, however, we discontinued ASCT when ASCT studies elsewhere [18–20] showed no survival advantage compared to the earlier MSKCC non-ASCT program using 3F8 without cytokines [15].

In desisting from ASCT, we reasoned that disease control could be achieved with a program encompassing: 1) more dose-intensive induction [1] compared to the CCG study [17] (a subsequent trial confirmed an advantage for increased dose-intensity [21]); 2) more potent anti-G_D2_ immunotherapy, by adding GM-CSF [22, 23], compared to the prior use of 3F8 without cytokines [13–15]; 3) local RT to the primary site in all patients [16, 24]; and 4) isotretinoin [17]. Additional considerations were hypothetical: low likelihood of ASCT agents ablating NB that had survived exposure (during induction) to high doses of identically- or similarly-acting chemotherapy; therapeutic advantage of earlier use of anti-G_D2_ mAbs by not having to wait for recovery from acute toxicities of ASCT; and avoiding the risk of infusing occult NB cells in the peripheral blood stem cells (a subsequent trial showed no survival advantage with purging [3]).

After 2003, two reports described randomized ASCT trials for HR-NB; each showed an advantage for ASCT [25, 26]. We, nevertheless, adhered to a non-ASCT program because of what we perceived as drawbacks in those trials: 1) their non-ASCT arms were at a distinct disadvantage, receiving no maintenance therapy in the British study (conducted 1982–1985) [25] and only oral cyclophosphamide in the German study (conducted 1997–2002) [26]; 2) the results - including those in a large successor European study (conducted 1990–1999) using the British model [21] - were not better than our initial non-ASCT program [15]; and 3) treatment was suboptimal by more recent standards, given the absence or irregular use of local RT, isotretinoin, and anti-G_D2_ mAb [21, 25, 26].

Since 2003, HR-NB patients referred to MSKCC were eligible for 3F8/GM-CSF with or without prior ASCT. We therefore accrued a study population of two groups treated during the same period and whose consolidative therapy, aside from ASCT, was identical – namely, 3F8/GM-CSF + isotretinoin and local RT. We analyzed this experience biostatistically to learn if ASCT improved prognosis. Large study size allowed use of key prognosticators including *MYCN*, minimal residual disease (MRD) [27], *FCGR2A* polymorphisms [28, 29], and killer immunoglobulin-like receptor (KIR) genotypes of natural killer cells [29, 30]. We now report results.

## RESULTS

### Patient characteristics

The 170 study patients (consecutively enrolled 05/2003–03/2013) included 60 treated following ASCT and 110 treated following conventional chemotherapy. Clinical and biological features that were not significantly different between these two groups included stage, age at diagnosis, *MYCN*, induction regimen [1–3, 21, 26, 31–33], MRD findings, *FCGR2A* allotypes, and missing KIR ligands (Table 1). Significantly different features included time from 1st chemotherapy to 3F8; time from ASCT or last chemotherapy to 3F8; ultra-high-risk (UHR) status; and use of high-dose 3F8. Among the UHR patients, 2nd-line treatments to achieve 1st CR/VGPR before study entry included regimens with topotecan [4, 5, 34] or irinotecan [5, 6]. ASCT involved carboplatin-etoposide-melphalan (*n* = 38) [3] or other myeloablative regimens in single (*n* = 11) or tandem (*n* = 11) transplant programs using alkylators (busulfan, cyclophosphamide, melphalan, thiotepa) ± other agents ± total body irradiation (TBI) [31–33]. All patients received local RT to the primary site [16, 24].

**Table 1:** Clinical and biological characteristics

		ASCT Status		
	All Patients	Yes (*n* = 60)	No (*n* = 110)	*p*-value
Gender				
Female	62 (36)	20 (33)	42 (38)	0.6177
Male	108 (74)	40 (67)	68 (62)	
Stage 4^a^	159 (94)	55 (92)	104 (95)	0.687
Age at diagnosis (months)	34.1 (0–179.3)^b^	33.4 (5.5–90.1)	36.2 (0–179.3)	0.6598
MYCN				
Not Amplified	76 (45)	22 (37)	54 (49)	0.1865
Amplified	86 (51)	34 (57)	52 (47)	
Time from 1st chemo to 3F8	7.7 (3.1–23.6)^b^	8.5 (6.4–17.2)	6.1 (3.1–23.6)	< 0.001
Time from ASCT/last chemo to 3F8	1.5 (0.8–7.5)^b^	2.3 (1.2–7.5)	1.3 (0.8–5.4)	< 0.001
Induction regimens				
Children's Oncology Group^1–3^	149 (87)	48 (80)	101 (92)	0.078
Limited institutional^36, 37^	13 (8)	8 (13)	5 (5)	
European^26, 27^	8 (5)	4 (7)	4 (4)	
Ultra-High-Risk^c^				
No	115 (68)	47 (78)	68 (62)	0.039
Yes	55 (32)	13 (22)	42 (38)	
High-Dose 3F8				
No	148 (87)	58 (97)	90 (82)	0.0072
Yes	22 (13)	2 (3)	20 (18)	
MRD at study entry (pre-MRD)				
Negative	119 (70)	40 (67)	79 (72)	0.4892
Positive	51 (30)	20 (33)	31 (28)	
MRD after 2 cycles (post-MRD)				
Negative	144 (85)	50 (83)	94 (85)	0.4581
Positive	21 (12)	5 (8)	16 (15)	
HAMA				
No	38 (22)	11 (18)	27 (25)	0.442
Yes	132 (78)	49 (82)	83 (75)	
FcGR2a				
HH	47 (28)	16 (27)	31 (28)	0.9552
HR	85 (50)	31 (52)	54 (49)	
RR	38 (22)	13 (22)	25 (23)	
FcGR3a				
FF	24 (14)	5 (8)	19 (17)	0.1752
VF	85 (50)	35 (58)	50 (45)	
VV	61 (36)	20 (33)	41 (37)	
KIR 2DL1 missing ligand				
No	91 (54)	30 (50)	61 (55)	0.523
Yes	79 (46)	30 (50)	49 (45)	
KIR 2DL2 2DL3 missing ligand				
No	151 (89)	55 (92)	96 (87)	0.4539
Yes	19 (11)	5 (8)	14 (13)	
KIR 3DL1 missing ligand				
No	108 (64)	40 (67)	68 (62)	0.6177
Yes	62 (36)	20 (33)	42 (38)	

Abbreviations: ASCT, autologous stem-cell transplantation; chemo, chemotherapy; HAMA, human anti-mouse antibody; NB, neuroblastoma; MRD, minimal residual disease in bone marrow

a All non-stage 4 patients had *MYCN*-amplified stage 3 except for one non-ASCT patient who had *MYCN*-amplified stage 2B.

b median (range)

c Because of an incomplete response to induction, 2nd-line therapy was needed to achieve 1st CR/VGPR.

### Outcome

**Figure 1:**
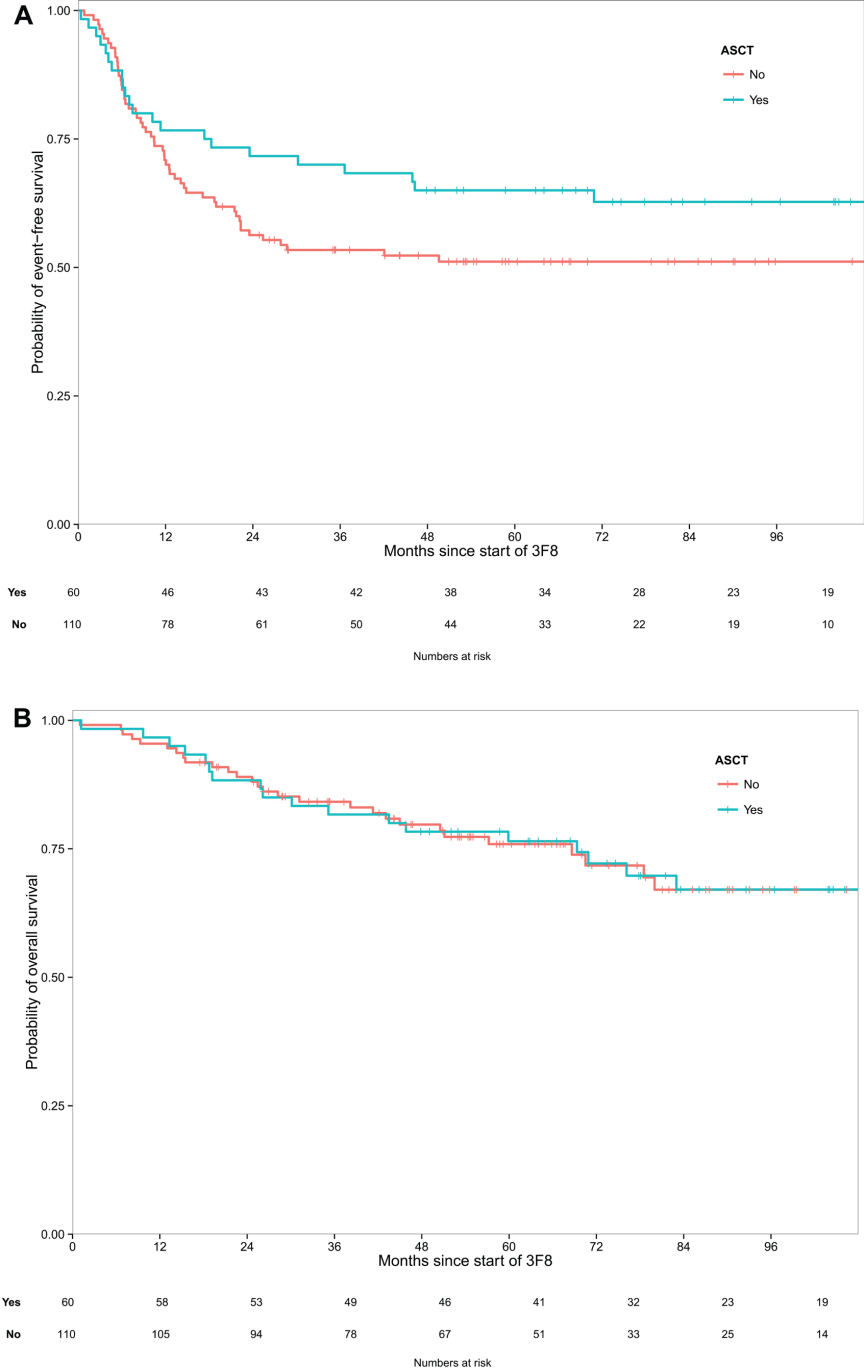
(**A**) A trend was seen toward better event-free survival post-transplant (*p* = 0.128). (**B**) Virtually identical overall survival was seen with consolidation following transplant or chemotherapy (*p* = 0.975).

Five-year rates for the ASCT cohort and the non-ASCT cohort were, respectively: EFS 65% (95% confidence interval [CI]: 54%–78%) *vs*. 51% (95% CI: 42%–62%) (log-rank *p* = 0.128), and OS 76% (95% CI: 66%–88%) *vs*. 76% (95% CI: 68%–85%) (log-rank *p* = 0.975) (Figure 1). Excluding the 55 UHR patients, five-year rates were: EFS 66% (95% CI: 54%–81%) *vs*. 52% (95% CI: 41%–66%) (*p* = 0.206), and OS 79% (95% CI: 68%–91%) *vs*. 77% (95% CI: 67%–89%) (*p* = 0.976). The median follow-up was 7.4 years (range 3.99 – 11.32) for surviving ASCT patients and 5.7 years (range 1.46 – 10.55) for surviving non-ASCT patients.

For EFS, the only two events other than PD were in ASCT patients: 1) acute leukemia 12 months from NB diagnosis and 4.5 months from study entry (NB subsequently relapsed); and 2) death from pulmonary fibrosis 78 months from NB diagnosis and 70 months from study entry.

As regards to OS, the non-ASCT cohort includes 14 patients in continuous 2nd CR/VGPR and off all therapy with long follow-up from relapse (42+ - to - 109+ months, median 71 months). The ASCT cohort includes three such patients (57+, 65+ and 72+ months).

### Univariate and multivariate analyses of prognostic factors

**Figure 2:**
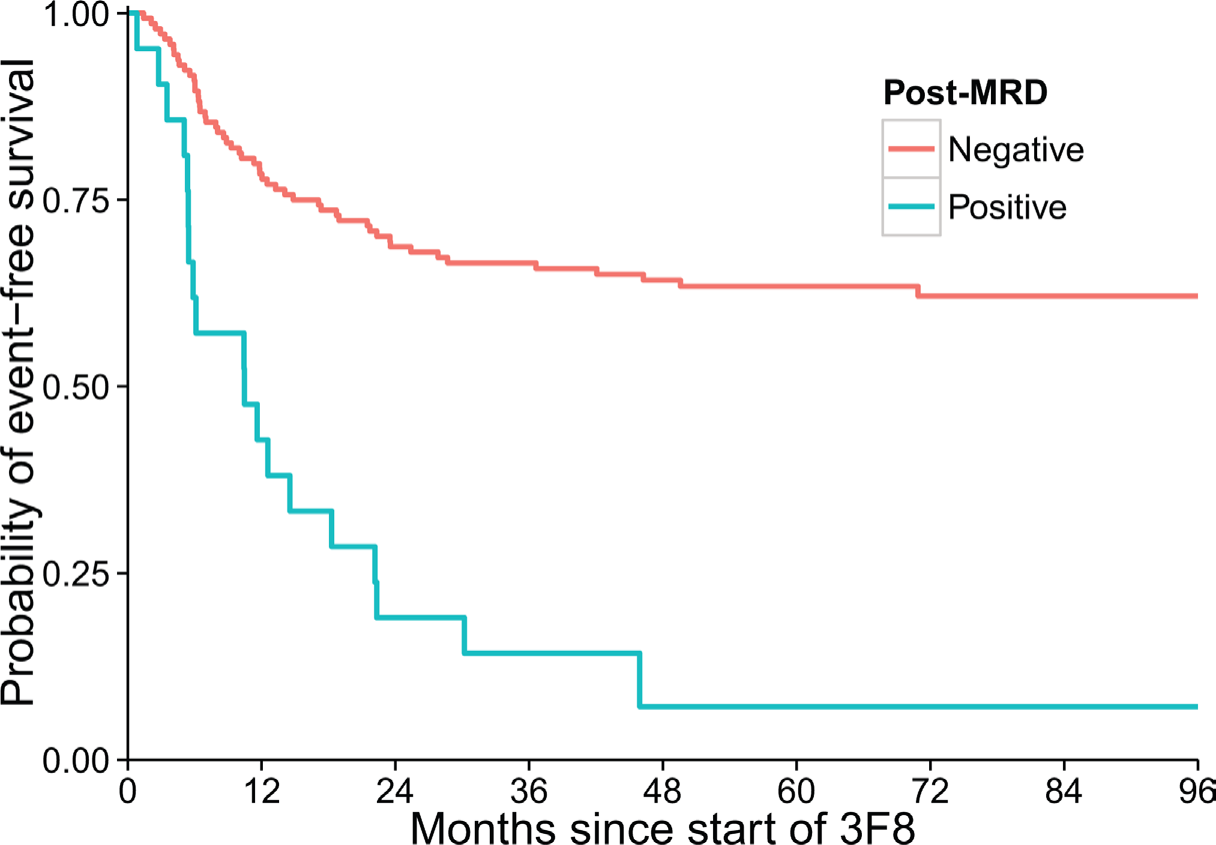
Strong association between minimal residual disease status after two cycles of 3F8/GM-CSF immunotherapy (post-MRD) and event-free survival of the 170 patients (*p* < 0.001)

In univariate analyses (Table 2), ASCT was not prognostic for EFS (hazard radio [HR] = 0.68, *p* = 0.13) or OS (*p* = 0.975). Post-MRD negativity was significantly associated with better EFS and OS (Figure 2). Longer time from 1st chemotherapy to 3F8 and longer time from ASCT or last chemotherapy to 3F8 were significant for better EFS (*p* = 0.012 and *p* = 0.022, respectively). HAMA as a time-dependent variable was not significant for EFS (*p* = 0.564) but marginally significant for OS (*p* = 0.058).

**Table 2:** Univariate analyses of patient and tumor characteristics for survival

	Event-free survival						Overall survival				
Variable	N used	N event	HR	95% Lower	95% Upper	*p*-value	N event	HR	95% Lower	95% Upper	*p*-value
Time from 1st chemo to 3F8	170	75	0.906	0.839	0.978	0.012	46	0.938	0.856	1.029	0.179
Time from ASCT/last chemo to 3F8	170	75	0.726	0.553	0.954	0.022	46	0.889	0.666	1.188	0.426
ASCT (Y *vs* N)	170	75	0.68	0.413	1.12	0.13	46	0.991	0.546	1.797	0.975
*MYCN*-amplified (Y *vs* N)	162	74	0.747	0.473	1.179	0.21	46	0.681	0.380	1.219	0.196
Ultra-High-Risk (Y *vs* N)	170	75	1.221	0.759	1.966	0.41	46	1.175	0.640	2.157	0.602
HAMA (Y *vs* N)*	170	75	1.169	0.687	1.989	0.564	46	0.536	0.279	1.022	0.058
High-Dose 3F8 (Y *vs* N)	170	75	1.507	0.791	2.871	0.212	46	2.220	0.902	5.467	0.083
Pre-MRD (Y *vs* N)	170	75	1.009	0.617	1.648	0.972	46	1.269	0.696	2.313	0.436
Post-MRD (Y *vs* N)*	165	72	4.997	2.894	8.627	< 0.001	43	4.304	2.232	8.301	< 0.001
FcGR2a											
HR *vs* HH	170	75	0.679	0.401	1.149	0.149	46	0.702	0.362	1.362	0.295
RR *vs* HH	170	75	0.865	0.469	1.594	0.642	46	0.748	0.336	1.666	0.477
FcGR3a											
VF *vs* FF	170	75	0.654	0.347	1.234	0.19	46	0.794	0.339	1.86	0.596
VV *vs* FF	170	75	0.646	0.332	1.258	0.199	46	0.862	0.357	2.08	0.741
KIR (Y *vs* N)	170	75	0.864	0.528	1.411	0.558	46	0.953	0.501	1.81	0.882
KIR 2DL1 (Y *vs* N)	170	75	0.973	0.617	1.534	0.907	46	1.37	0.766	2.45	0.288
KIR 2DL2 2DL3 (Y *vs* N)	170	75	0.754	0.346	1.643	0.478	46	0.525	0.163	1.693	0.281
KIR 3DL1 (Y *vs* N)	170	75	0.615	0.371	1.019	0.059	46	0.631	0.332	1.2	0.16
Trial (09–158/159 *vs*03–077)	170	75	1.370	0.735	2.552	0.321	46	1.883	0.764	4.642	0.169

* time-dependent variable

Since ASCT patients had longer time from 1st chemotherapy to 3F8 (Table 1), we undertook subset analyses. Among ASCT patients, time from 1st chemotherapy to 3F8 and time from ASCT to 3F8 were not significant for EFS (HR = 0.97 per month, *p* = 0.78 and HR = 0.97 per month, *p* = 0.86 respectively). In contrast, among non-ASCT patients, these variables were significant (HR = 0.91, *p* = 0.027, and HR = 0.49, *p* = 0.0146, respectively). When patients were split by the median time (7.75 months) from 1st chemotherapy to 3F8, ASCT was not significant for EFS (*p* = 0.44 for < 7.75 months group, *p* = 0.98 for ≥ 7.75 group). When patients were stratified by the median time (1.55 months) from ASCT or last chemotherapy to 3F8, ASCT was not significant (*p* = 0.134 for < 1.55 months group, and *p* = 0.61 for ≥ 1.55 months group). Based on these subset analyses, we concluded that univariate effect of ASCT on EFS (Table 2) was most likely a result of association between EFS and longer time from 1st chemotherapy or longer time from ASCT.

In the final multivariate analysis (Table 3), there was no significant impact of ASCT on EFS (*p* = 0.098) or OS (*p* = 0.655). As in the univariate analyses, post-MRD negativity correlated with significantly better EFS and OS (*p* < 0.001). Since time from 1st chemotherapy and time from ASCT or last chemotherapy were associated with ASCT, we fitted the multivariate model stratified by these variables dichotomized at median values. The effect of ASCT on EFS was not significant in a multivariate model adjusted for the post-MRD variable (*p* = 0.16 when stratified by time from 1st chemotherapy, and *p* = 0.21 when stratified by time from ASCT or last chemotherapy). The same held true for OS (*p* = 0.85 and *p* = 0.96, respectively).

**Table 3:** Multivariate analyses of patient and tumor characteristics for survival^a^

	Event-free survival				Overall survival			
Variable	HR	95% Lower	95% Upper	*p*-value	HR	95% Lower	95% Upper	*p*-value
ASCT (Y *vs* N)	0.630	0.364	1.089	0.098	0.866	0.460	1.629	0.655
Post-MRD (Pos *vs* Neg)^b^	4.596	2.602	8.118	< 0.001	4.295	2.226	8.285	< 0.001
Time from 1st chemo to 3F8	0.947	0.877	1.021	0.156	−	−	−	−

a Aside from ASCT, factors with *p* >.05 in univariate analyses were not included in the multivariate model.

b time-dependent variable

### Toxicity

As noted in previous reports [8, 35], 3F8/GM-CSF had manageable toxicities–hence, treatment was outpatient for the ASCT and non-ASCT groups. 3F8 caused grade 1–2 generalized pain and urticaria. Anaphylactoid reactions to 3F8 occurred on day 1 of cycle 2 in four patients, on day 3 of cycle 2 in one patient, and on day 1 of cycle 3 in one patient. Posterior reversible encephalopathy syndrome without sequelae developed in one patient in cycle 4. Common side-effects of isotretinoin were grade 1–2 dry skin and cheilitis. There were no long-term toxicities linked to protocol therapy.

## DISCUSSION

HR-NB patients whose consolidative therapy of 1st CR/VGPR included 3F8/GM-CSF + isotretinoin had similar EFS and near-identical OS whether these biological agents were administered following ASCT or conventional chemotherapy (Figure 1). In the multivariate analysis, ASCT was not significantly prognostic for either EFS or OS (Table 3). With its large cohort of non-ASCT patients, the current study's patient population may well be unique because ASCT has been part of all major studies since 2000 [9]. The experience, therefore, affords an opportunity not otherwise available to reassess whether ASCT (which is not standard for other extracranial solid tumors [36, 37]) should be routine for HR-NB. Revisiting this issue is especially timely given that the recent update of the landmark CCG study [17] showed no OS advantage with ASCT [38], and a recent meta-analysis found that ASCT for HR-NB did not improve OS [39].

The ASCT and non-ASCT patients were enrolled on study and treated during the same period by the same team and underwent the same surveillance (tests, schedule, duration) thereby avoiding biases seen with open-label trials [40]. Large study size (*n* = 170) allowed robust statistical analyses to assess relevant prognostic factors. An additional noteworthy point is that the two cohorts had similar clinical and biological characteristics, including *MYCN*, contemporary induction, and pre-MRD-positivity (Table 1). UHR status was significantly less common in the ASCT cohort – a clinical difference that might suggest a better outlook for the ASCT patients, but UHR was not prognostic (Table 2).

Because of the need to recover from ASCT toxicities, the time from initiation of induction chemotherapy and the time from ASCT or last chemotherapy to 3F8 were significantly longer for the ASCT cohort (Table 1). These differences could be considered selection bias that favored a superior outcome for the ASCT cohort because these patients would have more durable remissions whereas patients with early PD post-ASCT would not be eligible for 3F8/GM-CSF. The timing, however, was not prognostic in the multivariate analysis where the only variable significantly prognostic for both EFS and OS was MRD measured after two cycles of immunotherapy (similar to other studies [8, 35]) (Table 3).

Once viewed as a promising treatment for a range of poor-prognosis pediatric and adult extra-cranial solid tumors, ASCT became standard only for HR-NB [9, 36, 37]. The randomized CCG study [17], and the pilot tandem program that yielded excellent results [31], used TBI. Because of toxicity concerns, however, TBI was not used in recent national trials [2, 3, 32]. Assessments of ASCT ought to take into account differences in cytoreduction regimens. In the sole randomized trial of ASCT regimens for HR-NB reported to date [33], busulfan-melphalan was associated with significantly better EFS and OS compared to the widely-used carboplatin-etoposide-melphalan [3, 26]. The patients in our study were referred after these and tandem ASCT regimens [3, 31–33]. Irrespective of cytoreductive regimen, however, extensive experience over decades shows an uncertain benefit of ASCT for refractory NB [41, 42] – and the EFS and OS data in the current report would appear to undermine the rationale for the routine use of ASCT in HR-NB patients in 1st CR/VGPR in the contemporary era.

Another recent finding that raises uncertainty about ASCT for HR-NB is the lack of benefit from *ex vivo* purging [3]. In the COG randomized study of purging, occult NB contamination of peripheral blood stem cells was rare, suggesting that post-ASCT relapse could be attributed to the failure of the myeloablative regimen (carboplatin-etoposide-melphalan) to ablate the MRD that survived induction.

Advances in therapy since 2000 could account for the lack of survival advantage with ASCT in our experience. Thus, local control of soft tissue NB is excellent with dose-intensive chemotherapy, resection, and RT [16, 24, 43]; eradication of chemoresistant histologically-evident NB in BM is reliably achieved with anti-G_D2_ immunotherapy [35]; and novel salvage therapies have emerged. Regarding this last point, three ASCT and 14 non-ASCT patients are in continuous 2nd CR/VGPR and off all therapy with lengthy follow-up since relapse (42+–109+ months). HR-NB relapse has long been viewed as a systemic and ultimately lethal event; EFS has, therefore, been considered the most meaningful measure of efficacy. For curability of HR-NB, however, long-term OS may now supersede EFS endpoints given recent developments offering hope that the equivalence between relapse and lethality may no longer hold true. Thus, close monitoring [44] may now be detecting localized relapses [8], which might be controlled by surgery and/or focal RT, supplemented by systemic therapies that are non-cross-resistant with prior treatments. Examples include chemotherapy regimens [45, 46] and novel agents [47, 48]. Of note, a multi-modality salvage program centered on intrathecal ^131^I-labeled mAbs has yielded prolonged 2nd CR/VGPRs in patients with isolated relapse in the brain [49]. A bivalent vaccine combined with oral β-glucan has shown promise in consolidating 2nd CR/VGPR [50].

With cure of relapsed HR-NB being a realistic possibility, long-term OS gains increased importance. OS stands out as 1) the gold standard for evaluation of a treatment's efficacy; 2) the acid test for drug approval by the Food and Drug Administration; and 3) the driving force for advances in cancer therapeutics [40, 51, 52]. OS is 100% accurate for event and time, and it takes into account safety (toxic complications), which is a major concern with ASCT [53]. EFS is a surrogate endpoint for early assessment of efficacy, but its validity requires confirmation, either through correlation with OS or by meta-analysis [40, 51, 52]. This point is well illustrated by the disappearance of a long-term OS advantage for ASCT in the randomized CCG study [38] – an update reported after a meta-analysis had already identified no OS advantage with ASCT for HR-NB [39].

It would appear that the corrected results of the landmark CCG study [38], and the critical importance of long-term OS, support a reevaluation of ASCT for HR-NB. An additional consideration is that the randomized ASCT studies - conducted in 1982–1985 [25], 1991–1996 [17], and 1997–2002 [26] - have uncertain contemporary relevance, given the lower dose-intensity of their induction regimens and the absence or irregular use of local RT and anti-G_D2_ mAbs. The three randomized studies also preceded modern advances in salvage therapy and in the early detection of recurrent NB [44].

In conclusion, our experience, combined with a critical review of ASCT for HR-NB reaching back 30 years [39] and the loss of survival advantage with ASCT in a major study [38], suggests that ASCT may not improve outcome when local RT, anti-G_D2_ mAbs, and isotretinoin are used for consolidation after dose-intensive induction chemotherapy. A definitive confirmation of this welcome possibility would require a prospective randomized trial. Discontinuing ASCT for HR-NB would be consistent with the general consensus among pediatric oncologists that this highly toxic treatment in all other extracranial pediatric solid tumors is no longer recommended [36, 37].

## MATERIALS AND METHODS

Beginning in 2003, patients with HR-NB (stage 4 at age > 18 months or *MYCN*-amplified stage 2/3/4/4 s at any age) received 3F8/GM-CSF + isotretinoin to consolidate 1st CR/VGPR [54] documented following ASCT (patients referred to MSKCC) or conventional chemotherapy. UHR disease was defined as requiring 2nd-line therapy (because of an incomplete response to induction) to achieve this 1st CR/VGPR. This report concerns the HR-NB patients in 1st CR/VGPR enrolled on protocol 03–077 (NCT00072358) and the successor protocols 09–158 (NCT01183416) and 09–159 (NCT01183429). Major organ toxicity had to be grade < 2 by Common Terminology Criteria for Adverse Events Version 2.0, except absolute neutrophil count (ANC) ≥ 500/μl and platelet count ≥ 10,000/μl were acceptable. Informed written consents for treatments and tests were obtained according to MSKCC institutional review board rules.

### Protocol treatment

Immunotherapy cycles comprised priming doses of GM-CSF (Leukine, Immunex) × 5 days, followed by 3F8/GM-CSF × 5 days. 3F8 (prepared as described [55]) was intravenously infused over 30–60 minutes, with dosing at 20 mg/m^2^/day or, in a pilot study within the 09–158 / 09–159 protocols, 80 mg/m^2^/day (high-dose 3F8) for the 1st two cycles. GM-CSF was injected subcutaneously at 250 μg/m^2^/day for the five days of priming and the 1st two days of 3F8, and then increased to 500 μg/m^2^/day. GM-CSF was not given if the ANC was > 20,000/μl. These cycles were separated by 2-to-4-week intervals through cycle 4 and then by 6-to-8-week intervals through 24 months from study entry. Treatments were deferred if patients formed elevated human anti-mouse antibody (HAMA) titers (measured as described [56]). Isotretinoin was administered orally (× 6 courses, as described [17]) between cycles of 3F8, beginning post-cycle 2.

### Extent-of-disease evaluations and correlative studies

Disease status was assessed every 3 months for > 36 months by histology of BM aspirates and biopsies obtained from bilateral posterior and bilateral anterior iliac crests, ^123^I-metaiodobenzylguanidine (MIBG) scan, and computed tomography or magnetic resonance imaging of chest/abdomen/pelvis [44]. Disease status was defined by INRC [54], modified to incorporate ^123^I-MIBG findings. CR: no evidence of NB, including normal ^123^I-MIBG scan. VGPR: volume of primary mass reduced > 90%, normal ^123^I-MIBG scan, BM(−) by histology. PD: new lesion or > 25% increase in an existing lesion.

Quantitative reverse transcription-polymerase chain reaction was used to assess MRD, as described [8, 27], in BM before treatment (pre-MRD) and after two cycles of 3F8/GM-CSF (post-MRD). *FCGR2A* polymorphisms and KIR ligand mismatch were identified as described [8, 28, 30].

### Statistical analysis

The difference between clinical and biological features of the ASCT and non-ASCT cohorts was tested using Fisher's exact test for categorical variables and Wilcoxon rank-sum test for continuous variables. Event-free survival (EFS) was defined as time from start of 3F8 to PD, secondary malignancy, or toxic death, and was censored at last follow-up in the absence of these events. Overall survival (OS) was defined as time from start of 3F8 to death or last follow-up. The Kaplan-Meier method was used to calculate the probability of EFS and OS. The log-rank test was used to compare survival curves. Median follow-up was calculated using the Kaplan-Meier method and reversing the censoring indicator. Prognostic impact of clinical and biological features on EFS and OS was tested by univariate Cox proportional hazards regression. HAMA and post-MRD were evaluated as time-dependent variables. ASCT and variables significant in the univariate analysis (*p* < 0.05) were tested in the multivariate model. Time from the 1st dose of induction chemotherapy to 3F8 and time from ASCT or last dose of chemotherapy to 3F8 were correlated, so only one of them was chosen for multivariate model. Since these variables were correlated with ASCT, we also fitted multivariate models stratified by these variables dichotomized at their medians. All analyses were done using R version 3.0.2 (http://cran.r-project.org/).
